# Coinfections between Persistent Parasitic Neglected Tropical Diseases and Viral Infections among Prisoners from Sub-Saharan Africa and Latin America

**DOI:** 10.1155/2018/7218534

**Published:** 2018-11-06

**Authors:** Lilian Da Silva Santos, Hans Wolff, François Chappuis, Pedro Albajar-Viñas, Marco Vitoria, Nguyen-Toan Tran, Stéphanie Baggio, Giuseppe Togni, Nicolas Vuilleumier, François Girardin, Francesco Negro, Laurent Gétaz

**Affiliations:** ^1^Division of Prison Health, Geneva University Hospitals and University of Geneva, Geneva, Switzerland; ^2^Division of Tropical and Humanitarian Medicine, Geneva University Hospitals and University of Geneva, Geneva, Switzerland; ^3^Department of Control of Neglected Tropical Diseases, World Health Organization, Geneva, Switzerland; ^4^Department of HIV/AIDS & Global Hepatitis Program, World Health Organization, Geneva, Switzerland; ^5^Microbiology Laboratory, Unilabs, Coppet, Switzerland; ^6^Division of Laboratory Medicine, Department of Genetics and Laboratory Medicine, Geneva University Hospitals and Faculty of Medicine, Geneva, Switzerland; ^7^Medical Direction, Geneva University Hospitals and University of Geneva, Geneva, Switzerland; ^8^Department of Gastroenterology and Hepatology, Geneva University Hospitals, Geneva, Switzerland

## Abstract

In Swiss prisons, more than 70% of detained people are foreigners and over one-third originate from sub-Saharan Africa or Latin America. These two regions are endemic for various tropical diseases and viral infections, which persist after migration to nonendemic countries. Parasitic infections (schistosomiasis; strongyloidiasis) and cooccurrent viral infections (HIV, hepatitis B (HBV), and hepatitis C (HCV)) are especially of concern for clinical care but have been neglected in empirical research. These diseases often remain silent for years before causing complications, especially if they occur concomitantly. Our research aimed to study the prevalence rates and coinfections of two neglected tropical diseases, namely,* Strongyloides stercoralis* and* Schistosoma *sp. and viral infections among sub-Saharan Africans (SSA) and Latin Americans (LA) in Switzerland's largest pretrial prison. We carried out a cross-sectional prevalence study using a standardized questionnaire and serological testing. Among the 201 participants, 85.6% were SSA and 14.4% LA. We found the following prevalence ratios: 3.5% of HIV (4.1% in SSA, 0% in LA), 12.4% of chronic HBV (14.5% in SSA, 0% in LA), 2.0% of viraemic HCV (1.7% in SSA, 3.4% in LA), and 8.0% of strongyloidiasis (8.1% in SSA, 6.9% in LA). The serological prevalence of schistosomiasis among SSA was 20.3% (not endemic in Latin America). Two infections were simultaneously detected in SSA: 4.7% were coinfected with schistosomiasis and chronic HBV. Four other coinfections were detected among SSA: schistosomiasis-HIV, HIV-chronic HBV, HIV-HCV, and schistosomiasis-strongyloidiasis. To conclude, the high prevalence rates of persistent viral and parasitic infections and their potential coinfections among SSA and LA detained migrants highlight the need to implement control strategies and programs that reach people in detention centers in nonendemic countries.

## 1. Introduction

The global prevalence rate of human immunodeficiency virus (HIV), hepatitis B virus (HBV), and hepatitis C virus (HCV) approximates, respectively, 0.8%, 3.6%, and 1%, although their specific disease burden varies considerably between and within countries and regions [1–3]. These blood-borne infections remain a major health problem in most countries due to their associated morbidity and mortality [4]. Owing principally to long asymptomatic periods, the prevalence rates of chronic or persistent viral infections found in immigrants is related to the prevalence rates reported in their countries of origin where they spent part of their lives [5, 6].

Similarly, other infectious pathologies, including helminthiasis such as strongyloidiasis and schistosomiasis (both neglected tropical diseases, NTDs), can represent a persistent health problem among people who have migrated from endemic countries [7–9]. However, they have been neglected in epidemiological research. The research on the burden of infectious diseases among immigrants in Western countries rarely focuses on NTDs such as schistosomiasis and strongyloidiasis, probably because they are often a- or paucisymptomatic and because health professionals rarely consider these diseases [10].

### 1.1. Strongyloidiasis

Strongyloidiasis is endemic in tropical and subtropical regions. In immunocompetent persons, chronic intestinal infection usually remains paucisymptomatic for decades. In immunocompromised individuals, larvae can massively invade the gastrointestinal and pulmonary systems (hyperinfection syndrome) and other organs (disseminated strongyloidiasis). This invasion facilitates the translocation of enterobacteria leading to bacterial sepsis [11]. Hyperinfection syndrome and disseminated strongyloidiasis are severe conditions with high mortality rates (up to 87%) [12].

### 1.2. Schistosomiasis

Sub-Saharan Africa is the main endemic region for schistosomiasis: 93% of world's cases occur in this continent (mainly* S. mansoni* and* S. haematobium*) [13]. In Latin America, schistosomiasis (*S. mansoni*) is endemic only in certain areas of Brazil, Venezuela, Suriname, and some Caribbean islands [14]. Adult worms concentrate in the mesenteric (*S. mansoni* and* S. japonicum*) and perivesical (*S. haematobium*) veins, and the deposition of eggs in host tissues provokes inflammatory and fibrotic reactions. Infected patients can present abdominal pain, diarrhea, dysenteric syndromes (intestinal disease) and/or hematuria, dysuria, secondary infections, and ureteral fibrosis (genitourinary disease). Eggs can embolize the portal spaces of the liver, causing progressive fibrosis. One of the most feared complications is an upper gastrointestinal bleeding caused by esophageal varices [15].

### 1.3. Coinfections

Patients coinfected with viral and NTDs may have a more severe clinical course [5]. Coinfection of schistosomiasis with chronic HBV and HCV is associated with more severe forms of liver disease. Schistosomiasis can increase HIV transmission and accelerates HIV progression [16–25]. In HIV-infected patients, strongyloides infection can increase the risk of immune reconstitution syndrome, and in some circumstances, the risk of strongyloides hyperinfection, such as in patients treated with corticosteroids for toxoplasmosis or pneumonia caused by* Pneumocystis carinii* [26]. Data on prevalence rates of coinfections are needed to implement appropriate public health programs and improve awareness of health care staff within the prison context. Therefore, these coinfections should be at focus: patients with hepatopathy should be screened for schistosomiasis, as well as immunosuppressed patients for strongyloidiasis, in case of epidemiological risk factors for these helminths.

### 1.4. Detention Setting

In Europe, immigrants represent a high proportion of the correctional population. In 2012, non-European citizens accounted for 21% of the estimated 1.73 million people detained in the prisons of the 47 Member States of the Council of Europe. A large majority of foreign detainees originated from low-income countries. In Switzerland, inmates of non-Swiss nationality represent 73% of the correctional population, which is among the highest proportion of foreigners in detention in Western European countries. In the largest pretrial prison of Switzerland, 28% of detainees were SSA and 9% LA [27, 28]. People in prison represent a vulnerable and underserved population with often high morbidity but limited access to healthcare services [29, 30]. Therefore, detention settings are an opportunity to screen for, diagnose, and treat diseases with individual and sometimes public health (control of transmission) benefits [29, 31]. Furthermore, migrants from developing countries often lack access to health care, and therefore studies among people living in detention provide invaluable information for this underserved population.

The prevalence rates of blood-borne viruses are reported to be higher in prison settings than in the community. The main risk factors include history of injecting drug use, unprotected sexual intercourse, and unsafe tattooing [32]. There is limited knowledge on the relationship between migration to a nonendemic country and the epidemiologic profile of other infectious diseases, in particular persistent parasitic infections, among inmates from endemic regions.

Therefore, our study aimed to explore the epidemiologic profile of strongyloidiasis, schistosomiasis, HIV, HBV, and HCV among people from sub-Saharan Africa and Latin America who are detained in the pretrial detention center in Geneva, Switzerland.

## 2. Methods

### 2.1. Study Design, Population, and Setting

We used a cross-sectional study, which took place in 2015 at the pretrial detention center of Champ-Dollon (Geneva), the largest correctional center in Switzerland. The report of our study findings are based on the STROBE statement. Through an information campaign conducted in the detention center, all adults originating from Latin American or sub-Saharan African countries were invited to participate in the study. Interested individuals received information about the research and signed an informed consent form before study inclusion. A trained researcher administered a standardized questionnaire to each participant, whereupon a nurse collected a blood sample of circa 10 ml. We calculated the sample size (to decide the minimal number of participants that should be included in the study) according to the expected prevalence rates of strongyloidiasis and schistosomiasis, the core focus of the study. The expected prevalence rates for strongyloidiasis and schistosomiasis were estimated at 10% and 15% using a pilot study at Geneva correctional center [5] and prevalence rates among migrants in Europe [33–36]. With a +/-6% margin error and a confidence interval of 95%, the minimal sample sizes were, respectively, 97 and 137 participants for strongyloidiasis and schistosomiasis [37].

### 2.2. Questionnaire

Participants answered a standardized questionnaire in Spanish, Portuguese, English, or French (the interviewer was competent in these languages) (see Supplementary S1 Questionnaire). An initial short version of the questionnaire was offered to the first 30 participants, after which a longer version was finalized with additional questions. Questions explored sociodemographic characteristics (age, sex, country of birth, economic situation in the country of origin, and education level), sanitation conditions, and exposure to factors potentially related to viral infections (past and current injecting drug use, sexual behavior, body piercing other than ear-piercing and blood transfusion), and parasitic infections (walking barefoot in mud, swimming in lakes or rivers, and history of red urine during childhood).

### 2.3. Blood Sampling

Serological testing was performed at the Geneva university hospitals laboratory for viral diagnoses and at Unilabs laboratory (Coppet, Switzerland) for parasitic diagnoses. Serum samples were tested for HBV surface antigen (HBsAg), antibodies against HBV core antigen (anti-HBc), and surface antigen (anti-HBs), anti-HIV, and anti-HCV using commercial immunoenzymatic assays (Abbott Laboratories SA). Anti-HIV were systematically confirmed by INNO-LIA™ HIV I/II Score (Innogenetics, Belgium). Anti-HCV reactivity was systematically confirmed by INNO-LIA™ HCV Score (Innogenetics, Belgium) and quantitative HCV RNA measurements (Cobas Ampliprep TaqMan, v2.0, Roche Molecular Systems). The presence of these three positive tests signaled an active HCV infection. HBsAg positivity signaled a chronic HBV infection. The presence of anti-HBc antibodies and negative HBsAg indicated a resolved infection. Among participants with a serological pattern compatible with a resolved HBV (anti-HBc pos/HbsAg neg), occult HBV infections were not excluded. Since these cases are rare, underevaluation of chronic HBV prevalence rate in the study population would be very limited [38].

Screenings for strongyloidiasis and schistosomiasis were done using two ELISA commercial kits (Bordier Affinity Products SA, Switzerland). Bordier-ELISA kits for the diagnosis of schistosomiasis are reported to detect 94% of both* Schistosoma mansoni* and* Schistosoma haematobium* with a specificity of 94% in patients also coinfected with other helminths [39, 40]. Screening for schistosomiasis was performed using serum of all SSA and four LA (only those originating from endemic countries). The serologic test, Bordier-ELISA, for the detection of* Strongyloides stercoralis *has a sensitivity of 91% and a specificity of 94% among a composite population, which includes individuals from tropical areas who are coinfected with other parasitic infections [41]. All serological testing and screenings were performed according to the instructions from the manufacturers (Abbott, Innogenetics, Cobas, Bordier affinity products).

### 2.4. Clinical Management of Participants

All participants with confirmed HIV, HBV, HCV, schistosomiasis, and strongyloidiasis underwent a clinical evaluation. In cases of HIV, chronic HBV, and HCV, patients were referred to the Geneva university hospitals for specialized care. Antiparasitic treatments were given to participants who were positive for schistosomiasis (praziquantel 40mg/kg, once) and for strongyloidiasis (ivermectin 0.2mg/kg, once daily for two days). Vaccination against HBV was proposed to susceptible participants (i.e., anti-HBc and anti-HBs negative).

### 2.5. Statistical Analysis

The data were compiled into an Excel spreadsheet (see Supplementary S1 Dataset). We applied descriptive statistics using OpenEpi (version 3.01). Continuous variables were summarized as means and standard deviations and categorical variables were summarized as absolute and relative frequencies. Confidence intervals of prevalence ratios (95% CI) were calculated using Wilson's score interval. Chi-square and Fisher exact tests (when the assumptions of the chi-square test were not met) were used to explore the associations between infections and patients' characteristics. We also provided odd-ratios. Participants who answered “I don't know” or “I do not wish to answer” were not included in the related analyses. Due to the limited number of participants infected with HIV (n=7) and HCV (n=4), we did not investigate associations of these viral infections with potential risk factors. With regard to parasitic infections, association with swimming in lakes or rivers, history of red urine during childhood (for schistosomiasis), and walking barefoot in mud (for strongyloidiasis) were explored. A two-sided p-value less than to 0.05 was considered to be statistically significant.

### 2.6. Ethics Statement

The research project protocol was approved by the Ethics Committee of Geneva (number: 15-032; April 9, 2015).

## 3. Results

### 3.1. Participant Characteristics

Out of 403 inmates invited, 201 consented to participate in the study, answered the questionnaire, and completed blood analyses (participation rate: 49.9%). A total of 85.6% were SSA (23 countries) and 14.4% were LA (12 countries). A large majority of SSA participants originated from West or Central Africa (94.8%) (Table 1).

Table 2 summarizes the participants' sociodemographic characteristics. A total of 93.5% of participants were male, and 36.2% never attended school or had just reached primary school level. About a third reported having reading and/or writing difficulties. Reading inability or difficulties were more frequent among SSA participants (34.5%) than among LA (6.9%) (p<0.01).

With regard to factors potentially related to parasitic infections, the sanitation conditions before migration differed among participants from different regions: 96.5% of LA and 52.8% of SSA had indoor toilets (p<0.01) and 96.5% of LA reported having indoor tap water compared to 40.8% of SSA (p<0.01).

Regarding factors potentially related to viral infections, only one SSA participant (0.6%) and one LA (3.5%) reported a history of injecting drugs. More LA than SSA reported having tattoos (65.5%* versus* 11.8%, p<0.001), as well as body piercing (27.6%* versus* 0.7%, p<0.001). A history of blood transfusion was reported by 4.7% of the participants. In terms of sexual risk factors, 45.2% of males reported a history of transactional sex (against money or other favors). Half of the participants (49.8%) reported using condoms most of the time or always. Among the 156 male participants who reported being sexually active prior to detention, 98.7% reported being solely heterosexual.

### 3.2. Prevalence of Infection according to Origin

Table 3 presents the serological prevalence rates of strongyloidiasis, schistosomiasis, HIV, HBV, and HCV. A total of 3.5% of all participants were found to be HIV-positive: 4.1% among SSA and none among LA. Reflecting chronic or resolved HBV, 63.7% of participants had a positive anti-HBc: 72.7% among SSA, 10.3% among LA (p<0.001). The prevalence of chronic HBV was 12.4%: 14.5% among SSA, 0% among LA (p<0.03). Susceptibility to HBV (anti-HBc-negative, anti-HBs-negative) concerned 75.9% of LA and 18.6% of SSA (p<0.001), and 8.7% of SSA and 13.8% of LA had markers of HBV vaccinations (anti-HBc-negative, anti-HBs-positive). The prevalence rate of active HCV was 2.0%: 1.7% among SSA (2 cases of genotype 4 and one of genotype 2) and 3.4% among LA (1 case of genotype 1a) (p=0.93). A total of 42.9% (IC 95% 15.8-74.9) of participants who tested positive for HIV did not know their serostatus, 52.0% (IC 95% 33.5-70.0) of participants positive for chronic HBV, and 75.0% (IC 95% 24.2-98.8%) positive for HCV.

The serological prevalence rate of strongyloidiasis was 8.0%: 8.1% among SSA and 6.9% among LA (p=0.99). The serological prevalence rate of schistosomiasis, calculated among participants originating from sub-Saharan Africa (endemic area), was 20.3%.

### 3.3. Coinfections

Coinfections were detected only among SSA: 4.7% were coinfected with schistosomiasis and chronic HBV, 0.6% with schistosomiasis and HIV, 0.6% with HIV and chronic HBV, 0.6% with HIV and HCV, and 0.6% with schistosomiasis and strongyloidiasis.

### 3.4. Association with Sociodemographics, Lifestyle, and Exposure Factors

Among SSA participants, chronic or resolved HBV infection (anti-HBc-positive) was not significantly associated with age, education level, socioeconomic status, number of sexual partners, use of condom, or transactional sex (see S2 Table).

Among the four participants who were HCV positive, no one reported a history of injecting drugs or having tattoos or body piercing. Only one of them reported a history of blood transfusion.

Reporting history of “red urine during childhood” tended to be associated with schistosomiasis among SSA participants, although the association did not reach statistical significance (30%* versus* 17.9%; p=0.1). History of swimming in lakes or rivers was marginally associated with schistosomiasis (23.0%* versus* 10.8%; p=0.1) (see Supplementary S2 Table).

## 4. Discussion

This was the first study conducted in a prison setting designed to identify strongyloidiasis and schistosomiasis prevalence rates in Europe and their association with HIV, HBV, and HCV.

### 4.1. Epidemiology of Strongyloidiasis and Schistosomiasis

Our study in Swiss prison showed that a high proportion of migrants in detention from these endemic regions have parasitic NTDs (schistosomiasis seroprevalence: 20.3% among SSA, strongyloidiasis seroprevalence: 8% among SSA and LA). These findings corroborated the results from published literature on persistent parasitic diseases among migrants (schistosomiasis: 11–44%; strongyloidiasis: 7.5-42%), but focused on a neglected sub-group of migrants in Europe, i.e., detainees [13, 33–36, 42–47]. Cases previously reported in high-income country prisons are rare: only one schistosomiasis case in Spain [48] and no strongyloidiasis case since 1988, when Bourée et al. [26] described infections among Africans incarcerated in France. The high prevalence rates in our sample suggest mis- or underdiagnosis in this population, probably due to several factors. Strongyloidiasis and schistosomiasis are usually associated with no or a specific clinical presentation and require specialized diagnostic testing (coproparasitology and serology). Moreover, cases may be underreported in nonendemic countries, as helminthic infections are not subject to mandatory reporting.

### 4.2. Epidemiology of Blood-Borne Infections

The study population's prevalence ratio was 3.5% for HIV, 12.4% for chronic HBV, and 2.0% for viraemic HCV. These prevalence rates were higher than the respective prevalence rates in the Swiss general population (HIV: 0.2%, HBV: 0.18%, and HCV: 0.71%) [1, 49, 50]. These higher prevalence rates could be explained by exposure to and acquisition of these infections in the country of origin and the accumulation of unfavorable negative social determinants of health along the life course [32]. In our study population, a correlation with endemicity in the countries of origin was probable, as participants lived on average more than two-thirds of their lives in their country of origin.

The observed prevalence ratios of blood-borne infections among our participants were similar to the expected prevalence rates in the general population of the regions of origin. With regard to HIV, the expected prevalence rate is 3% in sub-Saharan Africa and 0.3% in Latin America and the Caribbean [2]. We found, respectively, prevalence ratios of 4.1% and 0% in our study.

For chronic HBV, the expected prevalence rate is 11.2% in West and Central Africa (14.5% in our study) and 0.8% in Latin America (0% in our study) [1]. Sexual health risk factors (number of sexual partners, history of activity with sex workers, and “nonuse” of condoms) were not significantly related to HBV in our study. It was possible that the sensitive nature of these questions led to an underreporting of these sexual behaviors. This underscored that asking these sensitive questions to migrant populations in prison may not always be useful in order to identify at-risk groups for HBV. The proportion of the study population susceptible to HBV (anti-HBc and anti-HBs negative) also varied according to origin. Only 9.4% of participants had markers of previous vaccinations, so catch-up vaccinations are recommended in prison settings [51].

The similarity between the prevalence rates in our study and the region of origin also applied to HCV. The global prevalence rate of viraemic HCV is estimated to be 1.5% in Western and Central sub-Saharan Africa in the general population [3]. We identified a prevalence rate of 1.7% among SSA participants, of whom 95% originated from Western and Central sub-Saharan Africa. In high-income countries, HCV seroprevalence in the correctional population is 10 to 20 times higher than that in the community, the main risk factor being intravenous drug use [32, 51]. Also, current HCV control programs in prisons mainly target people who inject drugs. The fact that none of the four HCV-positive participants reported a history of intravenous drug use reinforces the importance of considering origin as an independent risk factor among migrants. Acquisition of HCV may be attributable to unsafe blood transfusion, as reported by one participant, or to other well-known risk factors not investigated in the study, such as unsafe medical injections or exposure to contaminated blood, among others [52]. Genotypes 2 and 4 are related to 75% of HCV in Western and Central sub-Saharan Africa. These 2 serotypes are associated with only 13% of chronic HCV in Western Europe [3]. Two cases of serotypes 4 and one case of serotype 2 were diagnosed in the SSA participants (100% of infected SSA). This observation again indicated probable infections acquired in the country of origin prior to the migration process. Our findings suggested that control screenings should also target migrants without an intravenous drug use history originating from highly endemic countries. From a public health perspective, the high proportion of participants who tested positive for HIV, chronic HBV, or viraemic HCV and who did not know their serostatus confirms that incarceration represents an opportunity to screen for BBD in the study population. Prison is also an opportunity to develop public health campaigns designed to increase knowledge of transmission and control of infectious diseases, which may be useful upon release. Further studies should investigate more precisely the extent of awareness and health-seeking behaviors among inmates. A better understanding of their current knowledge and attention to such diseases would help to design effective interventions.

### 4.3. Epidemiology of Coinfections

A total of 4.7% of SSA participants had schistosomiasis and chronic HBV coinfection, and 0.6% had schistosomiasis and HIV coinfection. Detection and treatment of schistosomiasis are recommended for HIV-, HBV-, or HCV-positive persons with epidemiological risk factors. Indeed, schistosomiasis accelerates the development of liver fibrosis concomitantly with HBV or HCV infections and can increase the likelihood of HIV transmission, as well as accelerate the progression of HIV [16–25]. Treatment of schistosomiasis with praziquantel is well-tolerated and generally highly effective [15].

The correctional population is characterized by higher morbidity when compared to the host community [30] and special attention should be given to migrants from strongyloidiasis-endemic countries to detect and manage comorbidities that may precipitate hyperinfection syndrome/disseminated strongyloidiasis. These comorbidities include malignancies, human T-lymphotropic virus type 1 infection, diabetes mellitus, alcohol abuse, and severe malnutrition, in addition to the regular use of corticosteroids. Moreover, HIV infection predisposes individuals to hyperinfection syndrome/disseminated strongyloidiasis under certain conditions (e.g., immune reconstitution syndrome or opportunistic infections requiring corticoid prescription) [36, 53–55]. Therefore, coinfections and comorbidities should be at focus, because they have a severe burden of disease. Furthermore, treatment of uncomplicated strongyloidiasis is simple and highly effective and can be potentially life-saving before immunosuppressive therapy [56].

Two of the participants who were HIV-positive and were not injecting drug users had concomitant infections: one with HBV (HIV/HBV coinfection) and the other with HCV (HIV/HCV coinfection). Thus, these coinfections were not frequent but could be clinically important. HIV accelerates the natural course of HBV and HCV infection, facilitating faster liver fibrosis and malignancy [57]. Early diagnosis and treatment of coinfections are critical. In the case of an HIV/HBV coinfection, combination antiretroviral therapy (cART), which includes active agents against both HIV and HBV (HBV-active cART), significantly reduces liver-related mortality rates [58]. In the case of an HIV/HCV coinfection, direct-acting antiviral-based regimens against HCV are highly effective in HIV patients and can prevent the HIV/HCV-mediated acceleration of liver disease, which is a leading cause of morbidity and mortality [59].

### 4.4. Strengths and Weaknesses

First, the relatively small sample size limited the extension of statistical analyses to test associations between variables. However, the main study objective was not to confirm well-known risk factors of viral and parasitic diseases, but to investigate at a descriptive level the prevalence rates of selected parasitic NTDs and common viral infections. Second, the relatively low participation rate may have induced some degree of selection bias: questions about sex and sexual behavior are sensitive and many African Muslim participants refused to answer such questions; individuals of Muslim faith refused blood sampling during Ramadan; and the interviewer was a woman (for a largely male population). Therefore, there might be an underestimation of the prevalence rates of viral infections. We did not expect any influence on the prevalence rates of NTD. A third shortcoming was that the small sample size of prisoners having blood-borne and NTDs may have led to a lack of power when analyzing their associations with risk factors. Further studies using larger sample sizes should test whether migrant status and risk factors are cumulative risk factors for these diseases. In high-income countries, it would be useful to investigate whether the prevalence rates of persistent viral and NTDs among migrants in the general population correspond to that of incarcerated migrants.

## 5. Conclusion

The prevalence rates of viral infections, strongyloidiasis, and schistosomiasis were worryingly high in the studied prison population. Therefore, persistent parasitic NTDs should no longer be overlooked in custodial settings hosting migrants from endemic regions. Control efforts should target migrants originating from highly endemic countries. Indeed, correctional settings offer an opportunity to implement infectious disease control programs for a vulnerable population who has limited access to health care. Early diagnoses and treatments limit complications and costs for both patients and the host community. Therefore, detention settings must be part of the agenda for achieving target 3.3 of the United Nations Sustainable Development Goals, as it aims to end the NTDs epidemics and combat hepatitis among others.

## Figures and Tables

**Table 1:** Participants' countries of origin, Champ-Dollon detention center, Geneva, Switzerland, 2014-2015.

**Countries**	n=201

**Sub-Saharan Africa **	**172**

Western Africa^a^	154

Central Africa^b^	9

Eastern Africa^c^	6

Southern Africa^d^	3

**Latin America **	**29**

South America^e^	18

Central America^f^	11

^a^Guinea-Conakry (49), Nigeria (27), Guinea-Bissau (23), Gambia (21), Senegal (12), Ivory-Coast (5), Liberia (4), Mali (4), Niger (3), Sierra-Leon (3), Benin (1), Burkina-Faso (1), and Capo-Verde (1).

^b^Cameroon (3), RDC (3), Congo-Brazzaville (1), Gabon (1), and Central Africa Rep (1).

^c^Soudan (4), Somalia (1), and Ethiopia (1)

^d^Angola (2) and Tanzania (1)

^e^Bolivia (4), Chile (3), Colombia (3), Peru (3), Brazil (2), Venezuela (2), and Paraguay (1).

^f^Dominican Republic (7), Cuba (1), Jamaica (1), Mexico (1), and Nicaragua (1).

**Table 2:** Sociodemographic characteristics and factors potentially related to viral and parasitic infections, Champ-Dollon detention center, Geneva, Switzerland, 2014-2015.

**Characteristics**	**TOTAL** (n=201)	**African** (n=172)	**Latinos** (n=29)
**Age**			

mean in years (SD)	32.2 (± 9.4)	31.2 (±8.5)	38.4 (±11.8)

**Gender**			

Male	188 (93.5%)	163 (94.8%)	25 (86.2%)

Female	13 (6.5%)	9 (5.2%)	4 (13.8%)

**Residence in country of origin**			

Urban	146 (72.6%)	123 (71.5%)	23 (79.3%)

Rural	55 (27.4%)	49 (28.5%)	6 (20.7%)

**Years living in Europe**			

Mean in years (SD)	10.2 (7.7)	9.7 (7.1)	13.4 (9.0)

**Education level** ^**1**^			

Never attended school	25 (14.6%)	25 (17.6%)	0 (0%)

Primary	37 (21.6%)	33 (23.2%)	4 (13.8%)

Secondary	95 (55.5%)	77 (54.2%)	18 (62.1%)

University	14 (8.2%)	7 (4.9%)	7 (24.1%)

**Self rated socio-economical class** ^**1,2**^			

High	3 (1.8%)	3 (2.2%)	0 (0%)

Middle	112 (67.5%)	88 (64.2%)	24 (82.8%)

Low	51 (30.7%)	46 (33.6%)	5 (17.2%)

**Reading capacity** ^**1**^			

Don't know how to read	22 (12.9%)	22 (15.5%)	0 (0%)

Read with difficulty	29 (17.0%)	27 (19.0%)	2 (6.9%)

Read without difficulty	120 (70.1%)	93 (65.5%)	27 (93.1%)

**Writing capacity** ^**1**^			

Don't know how to write	24 (14.0%)	24 (16.9%)	0 (0%)

Write with difficulty	29 (17.0%)	26 (18.3%)	3 (10.3%)

Write without difficulty	118 (69.0%)	92 (64.8%)	26 (89.7%)

**Inside house lavatory**			

Yes	103 (60.2%)	75(52.8%)	28 (96.6%)

No	68 (39.8)	67 (47.2%)	1 (3.4%)

**Indoor tap water**			

Yes	86 (50.3%)	58 (40.8%)	28 (96.6%)

No	85 (49.7%)	84 (59.2%)	1 (3.4%)

**Injecting drugs** ^**1**^			

Yes	2 (1.2%)	1 (0.7%)	1 (3.5%)

No	169 (98.8%)	141 (99.3%)	28 (96.5%)

**Tattooing** ^**1**^			

Yes	36 (21.1%)	17 (12.0%)	19 (65.5%)

No	135 (78.9%)	125 (88%)	10 (34.5%)

**Body piercing** ^**1**^			

Yes	9 (5.3%)	1 (0.7%)	8 (27.6%)

No	162 (94.7%)	141 (99.3%)	21 (72.4%)

**Number of sexual partners** ^**1,3**^			

0	3 (1.5%)	3 (1.7%)	0

1-5	44 (27.9%)	41 (31.8%)	3 (10.7%)

6-20	55 (34.4%)	43 (32.5%)	12 (42.9%)

≥21	58 (36.2%)	45 (34.1%)	13 (46.4%)

**History of transactional sex** ^**1,4**^			

Yes	70 (45.2%)	59 (45.4%)	11 (44.0%)

No	85 (54.8%)	71(54.6)	14 (56%)

**Use of condom** ^**1**^			

Never- sometimes	85 (50.6%)	68 (48.9%)	17 (58.6%)

Most of times- always	83(49.4%)	71 (51.1%)	12 (41.4%)

**Sexual behaviour** ^**1,4**^			

Reported sexual contact with same-sex partner/s'	2 (1.3%)	1(0.8%)	1(4.0%)

Reported sexual contact only with opposite-sex partners'	154 (98.7%)	130 (99.2%)	24(96%)

**History of blood transfusion** ^**1**^			

Yes	8(4.6%)	7 (4.9%)	1(3.4%)

No	164 (95.4%)	136 (95.1%)	28 (96.6%)

**Walk in mudin country of origin** ^**1**^			

Never or rarely	119 (69.6%)	98 (69.0%)	21 (72.4%)

Often	52 (30.4%)	44 (31.0%)	8 (27.6%)

**Swim in lakes or rivers in country of origin**			

Never	46 (23.0%)	37 (21.5%)	9 (32.1%)

Rarely, sometimes or often	154 (77.0%)	135 (78.5%)	19 (67.8%)

^1^Not included in the initial questionnaire which was administered to the first 30 participants. ^2^5 participants responded “I don't know”. ^3^No response for 11 participants (answer: I do not wish to respond). ^4^Including only males with sexual activity.

**Table 3:** Serological prevalence of strongyloidiasis, schistosomiasis, HIV, hepatitis B virus (HBV), and hepatitis C virus (HCV) among 201 participants from sub-Saharan Africa and Latin America, Champ-Dollon detention center, Geneva, Switzerland, 2014-2015.

	**TOTAL**		**Sub-Saharan Africans**		**Latino**		
	n=201	% (95% CI)	n=172	% (95% CI)	n=29	% (95% CI)	p-value (*χ*^2^)
Strongyloidiasis	16	8.0 (5.0-12.5)	14	8.1 (4.9-13.2)	2	6.9 (1.9-22.0)	0.99^2^

Schistosomiasis	-	-	35	20.3 (15.0-27.0)	-	Not endemic^1^	-

Anti-HIV+	7	3.5 (1.7-7.0)	7	4.1 (2.0-8.2)	0	0 (0-11.7)	0.66^2^

Chronic/resolved HBV (anti-HBc+)	128	63.7 (56.9-70.1)	125	72.7 (65.6-78.8)	3	10.3 (3.6-26.4)	**<0.001**

Chronic HBV	25	12.4 (8.6-17.7)	25	14.5 (10.0 -20.6)	0	0 (0-11.7)	**0.03** ^2^
(HBsAg+)							

Viraemic HCV	4	2.0 (0.8-5.0)	3	1.7 (0.6-5.0)	1	3.4 (0.6-17.2)	0.93^2^

^1^ In Latin America, transmission of schistosomiasis only in Brazil, Venezuela, and Suriname: none of the four participants originating from these countries were positive. ^2^ Fisher exact tests were performed.

## Data Availability

The dataset is available as a supplementary material of the study.

## Supplementary Materials

Supplementary Materials Supplementary S1 Questionnaire. Supplementary S1 Table: sociodemographics, lifestyle, exposure, and clinical factors associated with anti-Hbc in bivariate analysis, according to serological status, Champ-Dollon prison, Geneva, Switzerland, 2014-2015. Supplementary S2 Table: sociodemographics, lifestyle, exposure, and clinical factors associated with strongyloidiasis and schistosomiasis in bivariate analyses, according to serological status, Champ-Dollon detention center, Geneva, Switzerland, 2014-2015. Supplementary S1 Dataset.
