# Supplementary material for: Coinfections between Persistent Parasitic Neglected Tropical Diseases and Viral Infections among Prisoners from Sub-Saharan Africa and Latin America
Source: J Trop Med. 2018 Nov 6;2018:7218534. doi: 10.1155/2018/7218534 (PMC6247427; doi:10.1155/2018/7218534)
Supplement: Supplementary Materials — Supplementary S1 Questionnaire. Supplementary S1 Table: sociodemographics, lifestyle, exposure, and clinical factors associated with anti-Hbc in bivariate analysis, according to serological status, Champ-Dollon prison, Geneva, Switzerland, 2014-2015. Supplementary S2 Table: sociodemographics, lifestyle, exposure, and clinical factors associated with strongyloidiasis and schistosomiasis in bivariate analyses, according to serological status, Champ-Dollon detention center, Geneva, Switzerland, 2014-2015. Supplementary S1 Dataset. [file 7218534.f1.docx]

**S1 Table –** Socio-demographics, lifestyle and exposure factors associated with AntiHbc according to bivariate analyses among inmates originating from sub-Saharan Africa, Champ-Dollon prison, Geneva, Switzerland, 2014-15.

| **Characteristics** | **Anti-HBc (n=172)** | |  | **AgHbs (n=172)** | |  |
| --- | --- | --- | --- | --- | --- | --- |
|  | **Positive** | **Negative** | **p-value** (χ^2^)  **OR (95%IC)** | **Positive** | **Negative** | **p-value** (χ^2^)  **OR (95%IC)** |
| **Age** |  |  |  |  |  |  |
| <=30 | 62 (68.1%) | 29 (31.9%) | 0.16 | 11 (12.1%) | 80 (87.9%) | 0.33 |
| >30 | 63 (77.8%) | 18 (22.2%) | 0.6 (0.3-1.2) | 14 (17.3%) | 67 (82.7%) | 0.7 (0.3-1.6) |
| **Residence in country of origin** |  |  |  |  |  |  |
| Rural/Both | 33 (67.3%) | 16 (32.7%) | 0.32 | 11 (22.4%) | 38 (77.6%) | 0.06 |
| Urban | 92 (74.8%) | 31 (25.2%) | 0.7 (0.3-1.5) | 14 (11.4%) | 109 (88.6%) | 2.2 (0.9-5.4) |
| **Education level^1^** |  |  |  |  |  |  |
| Never/primary | 46 (79.3%) | 12 (20.7%) | 0.23 | 8 (13.8%) | 50 (86.2%) | 0.93 |
| Secondary/University | 59 (70.2%) | 25 (29.8%) | 1.6 (0.7-3.7) | 12 (14.3%) | 72 (85.7%) | 1.0 (0.4-2.5) |
| **Socio-economical status^1,2^** |  |  |  |  |  |  |
| Low | 34 (73.9%) | 12 (26.1%) | 0.92 | 7 (15.2%) | 39 (84.8%) | 0.88 |
| High-middle | 68 (74.7%) | 23 (25.3%) | 1.0 (0.4-2.2) | 13 (14.3%) | 78 (85.7%) | 1.1 (0.4-2.9) |
| **Number of sexual partners^1,3^** |  |  |  |  |  |  |
| <=10 | 45 (71.4%) | 18(28.6%) | 0.37 | 7 (11.1%) | 56 (88.9%) | 0.31 |
| >10 | 54 (78.3%) | 15 (21.7%) | 0.7 (0.3-1.5) | 12 (17.4%) | 57 (82.6%) | 0.6 (0.2-1.6) |
| **Use of condom^1^** |  |  |  |  |  |  |
| Never- sometimes | 48 (70.6) | 20 (29.4%) | 0.35 | 5 (7.3%) | 63 (92.7%) | **0.03** |
| Most of times- always | 55 (77.5) | 16 (22.5%) | 0.7 (0.3-1.5) | 14 (19.7%) | 57 (80.3%) | 0.3 (0.1-0.9) |
| **Transactional sex^1,4^** |  |  |  |  |  |  |
| Yes | 47 (79.7) | 12 (20.3%) | 0.39 | 10 (16.9%) | 49 (83.1%) | 0.49 |
| No | 52 (73.2) | 19 (26.8%) | 1.4 (0.6-3.3) | 9 (12.7%) | 62 (87.3%) | 1.4 (0.5-3.8) |
| **Blood transfusion^1^** |  |  |  |  |  |  |
| Yes | 6 (85.7%) | 1 (14.3%) | 0.85^5^ | 2 (28.6%) | 5 (71.4%) | 0.51^5^ |
| No | 99 (73.9%) | 35 (26.1%) | 2.1 (0.3-50.4) | 18 (13.3%) | 117 (86.7%) | 2.6 (0.3-14.1) |
| **Injecting drug use^1^** |  |  |  |  |  |  |
| Yes | 1 (100%) | 0 (0%) | 0.99^5^ | 0 (0%) | 1 (100%) | 0.99^5^ |
| No | 104 (73.8%) | 37 (26.2%) | - | 20 (14.2%) | 121 (85.8%) | - |
| **Tattoo^1^** |  |  |  |  |  |  |
| Yes | 11(64.7%) | 6 (35.3%) | 0.51^5^ | 3 (17.6%) | 14 (82.4%) | 0.88^5^ |
| No | 94 (75.2%) | 31 (24.8%) | 1.65 (0.5-4.8) | 17 (13.6%) | 108 (86.4%) | 1.4 (0.3-5.0) |

^1^Not asked to 30 participants (during pilot phase); ^2^ Don’t know: 5;

^3^ Do not wish to respond: 10; ^4^ Includes only male with sexual activity; ^5^Fisher exact tests were performed.

**S2 Table –**Socio-demographics, lifestyle, exposure, and clinical factors associated with strongyloidiasis and schistosomiasis in bivariate analyses, according to serological results, Champ-Dollon detention center, Geneva, Switzerland, 2014-2015.

| **Characteristics** | **Strongyloidiasis (n=201)** | | | **Schistosomiasis (n=172^1^)** | | |
| --- | --- | --- | --- | --- | --- | --- |
|  | **pos** | **neg** | **p-value** (χ^2^)  **OR (95%IC)** | **pos** | **neg** | **p-value** (χ^2^)  **OR (95%IC)** |
| **Origin** |  |  |  |  |  |  |
| sub-Saharan African | 14 (8.1%) | 158 | 0.99^5^ | 35 (20.3%) | 137 | - |
| Latin America | 2 (6.9%) | 27 | 1.2 (0.3-8.2) | - | - |  |
| **Age** |  |  |  |  |  |  |
| <=30 | 6 (6.1%) | 92 | 0.35 | 21 (23.1%) | 70 | 0.35 |
| >30 | 10 (9.7%) | 93 | 0.6 (0.2-1.7) | 14 (17.3%) | 67 | 1.4 (0.7-3.1) |
| **Gender** |  |  |  |  |  |  |
| Female | 1 (6.2%) | 12 | 0.99^5^ | 0 | 9 | 0.24 |
| Male | 15 (8%) | 173 | 0.96 (0.1-6.2) | 35 (21.5%) | 128 | - |
| **Residence in country of origin** |  |  |  |  |  |  |
| Rural/Both | 6 (10.9%) | 49 | 0.50^5^ | 12 (24.5%) | 37 | 0.39 |
| Urban | 10 (6.8%) | 136 | 1.7 (0.5-5.4) | 23 (18.7%) | 100 | 1.4 (0.6-3.1) |
| **Education level^2^** |  |  |  |  |  |  |
| Never/primary | 6 (9.7%) | 56 | 0.6 | 9 (15.5 %) | 49 | 0.30 |
| Secondary/University | 8 (7.3%) | 101 | 1.4 (0.4-4.1) | 19 (22.6%) | 65 | 0.6 (0.3-1.5) |
| **Self rated socio-economical class^2,3^** |  |  |  |  |  |  |
| Low | 2 (3.9%) | 49 | 0.27^5^ | 11 (23.9%) | 35 | 0.47 |
| High-middle | 12 (10.4%) | 103 | 0.35 (0-1.7) | 17 (18.7%) | 74 | 1.4 (0.6-3.2) |
| **Sanitation conditions** |  |  |  |  |  |  |
| Inside house lavatory | 8 (7.8%) | 95 | 0.80 | 15 (20%) | 60 | 0.92 |
| Outhouse or no lavatory | 6 (8.8%) | 62 | 0.9 (0.3-2.8) | 13 (19.4%) | 54 | 1.04 (04-2.4) |
| **Type of water^2^** |  |  |  |  |  |  |
| Inside house tap water | 7 (8.1%) | 79 | 0.98 | 10 (17.2%) | 48 | 0.54 |
| Other ^4^ | 7 (8.2%) | 78 | 0.99 (0.3-3.1) | 18 (21.4%) | 66 | 0.8 (0.3-1.8) |
| **Walk in the mud barefoot/open shoes** |  |  |  |  |  |  |
| Rarely - often-very often | 1 (3.2%) | 30 | 0.47^5^ | 1 (27.3%) | 23 | 0.06^5^ |
| Never | 13 (9.3%) | 127 | 0.3(0.0-2.0) | 26 (16.3%) | 92 | 0.15 (0.0-1.1) |
| **Swim in lakes or rivers** |  |  |  |  |  |  |
| Rarely-sometimes-often | 13 (8.8%) | 135 | 0.52^5^ | 31 (23.0%) | 104 | 0.10 |
| Never | 2 (4.3%) | 44 | 2.1 (0.5-20) | 4 (10.8%) | 33 | 2.5 (0.9-8.7) |
| **Red urine during childhood** |  |  |  |  |  |  |
| Rarely-often | - | - |  | 12 (30.0%) | 28 | 0.10 |
| Never |  |  |  | 22 (17.9%) | 101 | 2.0 (0.8-4.4) |

^1^ All Latin American participants (29) not included (schistosomiasis not endemic in Latin America, apart few areas in Brazil, Venezuela and Suriname); ^2^ Not asked to 30 participants (during pilot phase); ^3^ Five participants respond “don’t know”; ^4^ Sealed well/outhouse tap water or open well or river. ^5^ Fisher exact tests were performed.

Patient N° : _ _ _ _ _

| **1) Surname : _ _ _ _ _ _ _ _ _ _ _ _ _ _ _ _ _ _** | **2) First name : _ _ _ _ _ _ _ _ _ _ _ _ _ _ _ _ _** |
| --- | --- |
| **3) Date of birth : _ _ / _ _ / _ _ _ _** | **4) Country of origin : _ _ _ _ _ _ _ _ _ _ _ _ _** |
| **5) Year of arrival in Europe: _ _ _ _** | **Gender:** male female |
| **6) Have you lived in other countries in Africa or Latin America** (other than your country of origin?)**:**  A. Yes B. No | |
| **If yes, where ? Duration**  Month Year Month Year | |
| **Country 1) _ _ _ _ _ _ _ _ _ _  _ _ from : _ _ _ _ _ _ _ _ to : _ _ _ _ _ _ _ _** | |
| **Country 2) _ _ _ _ _ _ _ _ _ _  _ _ from : _ _ _ _ _ _ _ _ to  : _ _ _ _ _ _ _ _** | |
| **Country 3) _ _ _ _ _ _ _ _ _ _  _ _ from : _ _ _ _ _ _ _ _ to : _ _ _ _ _ _ _ _** | |
| **Country 4) _ _ _ _ _ _ _ _ _ _  _ _ from : _ _ _ _ _ _ _ _ to : _ _ _ _ _ _ _ _** | |
| **7) Since arriving in Europe, have you ever left Europe on vacation or to work?**  A. Yes B. No  If yes : Where ? **_ _ _ _ _ _ _ _ _ _  _ _**  and when ? **_ _ _ _ _ _ _ _ _ _  _ _**  Where  ? **_ _ _ _ _ _ _ _ _ _  _ _**  and when ? **_ _ _ _ _ _ _ _ _ _  _ _** | |
| **8) In your country of origin, did you live:**  A. Mainly in a town B. Mainly in the countryside C. Both | |
| **9) In your country of origin, what type of toilet did you use most frequently**  A.Toilet in the home with running water B. Outside toilet with running water  C. Outside latrines without running water D. No toilet  E. Other : _ _ _ _ _ _ _ _ _ | |
| **10) In your country of origin, where did you normally get your drinking water?**  A. Tap water inside the home B. Closed well with pump or outside tap  C. Open well D. River E. Other: _ _ _ _ _ _ _ _ _ _ _ _ | |
| **11) During the rainy season in your country of origin, did you usually walk about outside**  A. Barefoot B. Wearing open footwear C. Wearing enclosed footwear | |
| **12) When you lived in your country of origin, did you walk barefoot or in sandals through mud?**  A. Never B. Rarely C. Often D. Very often | |
| **13) In your country of origin, how would you characterize your socioeconomic level ?**  A. High B. Middle class C. Low socioeconomic level D. Don’t know | |
| **14) What is your level of education?**  □ A. *Incomplete primary schooling* □ B. *Completed primary schooling*  □ C. *Incomplete secondary schooling* □ D. *Completed secondary schooling*  □ E. *Incomplete* college/school leaving examination □ F.*Completed* college/school leaving examination  □ G. *Technical education after secondary school* □ H. *University* | |
| **15) Reading**  □ A. I do not know how to read □ B. I can read with difficulty □ C. I can read without difficulty | |
| **16) Writing**  □ A. I do not know how to write □ B. I can write with difficulty □ C. I can write without difficulty | |
| **17) Have you ever injected drugs (for example heroin or cocaine) intravenously?**  A. Yes B. No C. Don’t know | |
| **18a) Have you got one or more tatoos?**  A. Yes B. No  **18b) If yes, were they all applied using sterile equipment?**  A. Yes B. No C. Don’t know | |
| ***Answer questions 19 and 20 only if you are from sub-Saharan Africa***  **19) When you lived in your country of origin, did you swim or wade in lakes or rivers?**  A. Often B. Occasionally C. Rarely D. Never  **20) When you were young, did your urine sometimes appear red?**  A. Yes, sometimes B. Yes, rarely C. No, never D. Don’t know | |

| **Risks associated with HIV and hepatitis** |
| --- |
| **21) Approximately how many sexual partners have you had in your life?**  A. 0 B. 1-5 C. 6-10 D. 11-20 E. 20+ E. I do not wish to respond |
| **22) Your sexual partners are :**  A. Men B. Women C. Both |
| **23) When you have sex, do you use condoms?**  A. Always B. Often C. Occasionally D. Never |
| **24) Have you ever paid someone to have sex, or had sex in exchange for drugs, food or other things?**  A. Yes B. No B. I do not wish to respond |
| **25) Have you had sex with someone infected with:**  A. Hepatitis B B. Hepatitis C C. HIV D. Don’t know |
| **26) Have you ever had a blood transfusion?**  A. Yes B. No C. Don’t know |
| **27) Do you have a body piercing (other than in your ears)?**  A. Yes B. No |
| **27) Do you know if you are infected by hepatitis B, hepatitis C or HIV ?**  A. Yes B. No C. I do not know  **Il yes, which one ? :** A. Hepatitis B B. Hepatitis C C. HIV |

**Comments  _ _ _ _ _ _ _ _ _ _ _ _ _ _ _ _ _ _ _ _ _ _ _ _ _ _ _ _ _ _ _ _ _ _ _ _ _ _ _ _ _ _ _ _**

**_ _ _ _ _ _ _ _ _ _ _ _ _ _ _ _ _ _ _ _ _ _ _ _ _ _ _ __ _ _ _ _ _ _ _ _ _ _ _ _ _ _ _ _ _ _ _ _ _ _ _ _ _ _ _ _ _ _ _ _ _ _ _ _ _ _ _ _ _ _ _ _ _ _ _ _ _ _ _ _ __ _ _ _ _ _ _ _ _ _ _ _ _ _ _ _ _ _ _ _ _ _ _ _ _ _ _**
